# Multiple Mechanisms Mediate Resistance to Sorafenib in Urothelial Cancer

**DOI:** 10.3390/ijms151120500

**Published:** 2014-11-07

**Authors:** Judith Knievel, Wolfgang A. Schulz, Annemarie Greife, Christiane Hader, Tobias Lübke, Ingo Schmitz, Peter Albers, Günter Niegisch

**Affiliations:** 1 Department of Urology, Heinrich-Heine-University, Moorenstr. 5, Düsseldorf D-40225, Germany; E-Mails: judith.knievel@hhu.de (J.K.); wolfgang.schulz@hhu.de (W.A.S.); annemarie.koch@hhu.de (A.G.); christiane.hader@hhu.de (C.H.); urologie@uni-duesseldorf.de (P.A.); 2 Helmholtz-Zentrum für Infektionsforschung, Inhoffenstr. 7, Braunschweig D-38124, Germany; E-Mails: tobias.luebke@helmholtz-hzi.de (T.L.); ingo.schmitz@helmholtz-hzi.de (I.S.); 3 Institute for Molecular and Clinical Immunology, Otto-von-Guericke-University, Leipzigerstr. 44, Magdeburg D-39120, Germany

**Keywords:** tyrosine kinase inhibitor, urothelial cancer, mitogen activated protein kinase (MAPK) signaling, apoptosis

## Abstract

Genetic and epigenetic changes in the mitogen activated protein kinase (MAPK) signaling render urothelial cancer a potential target for tyrosine kinase inhibitor (TKI) treatment. However, clinical trials of several TKIs failed to prove efficacy. In this context, we investigated changes in MAPK signaling activity, downstream apoptotic regulators and changes in cell cycle distribution in different urothelial cancer cell lines (UCCs) upon treatment with the multikinase inhibitor sorafenib. None of the classical sorafenib targets (vascular endothelial growth factor receptor 1/-receptor 2, VEGFR1/-R2; platelet-derived growth factor receptor α/-receptor β, PDGFR-α/-β; c-KIT) was expressed at significant levels leaving RAF proteins as its likely molecular target. Low sorafenib concentrations paradoxically increased cell viability, whereas higher concentrations induced G1 arrest and eventually apoptosis. MAPK signaling remained partly active after sorafenib treatment, especially in T24 cells with an oncogenic HRAS mutation. AKT phosphorylation was increased, suggesting compensatory activation of the phosphatidylinositol-3-kinase (PI3K) pathway. Sorafenib regularly down regulated the anti-apoptotic myeloid cell leukemia 1 (Mcl-1) protein, but combinatorial treatment with ABT-737 targeting other B-cell lymphoma 2 (Bcl-2) family proteins did not result in synergistic effects. In summary, efficacy of sorafenib in urothelial cancer cell lines appears hampered by limited effects on MAPK signaling, crosstalk with further cancer pathways and an anti-apoptotic state of UCCs. These observations may account for the lack of efficacy of sorafenib in clinical trials and should be considered more broadly in the development of signaling pathway inhibitors for drug therapy in urothelial carcinoma.

## 1. Introduction

Accounting for more than 300,000 newly diagnosed patients every year, bladder cancer is the seventh most common cancer worldwide [1]. In most regions of the world, the majority of bladder cancer patients present with urothelial cancer. Urothelial cancer (UC) can be clinically categorized into two major subtypes. About one third of the patients suffer from muscle-invasive cancers, which are prone to metastatic spread and carries a poor prognosis (20% survival after 5 years). Approximately two thirds of patients present with tumors restricted to the bladder mucosa, which very frequently develop metastases and have a much better prognosis (survival >90% after five years) [2].

Both subtypes are also distinguishable at the molecular and genetic level [3,4]. Muscle-invasive UC is commonly genetically unstable with genetic and epigenetic changes especially in cell cycle regulation and checkpoints (e.g., RB1, CDKN2A/p16^INK4A^, Cyclins, p53, ATM). These changes are rarer in non-muscle-invasive UC. These tumors are often genetically stable and commonly contain mutations leading to overactivity of mitogen activated protein kinase (MAPK) and phosphatidylinositol-3-kinase (PI3K) signaling pathways, e.g., oncogenic mutations in fibroblast growth factor receptors 3 (FGFR3), PIK3CA and HRAS. However, a fraction of morphological superficial UCs likewise presents with altered cell cycle control and possess a potential for invasion and metastatic spread. Conversely, mutations in *FGFR3*, *PI3KCA* and *RAS* genes are also observed in muscle-invasive cancer as well as overexpression or mutations of the endothelial growth factor receptor (EGFR), ErbB-2 and ErbB-3 receptor tyrosine kinases and inactivation of pathway inhibitors such as tuberous sclerosis complex 1 (TSC1) or PTEN [5,6,7,8,9]. These changes render UC potentially suitable for drugs targeting tyrosine kinases and signaling pathways stimulating proliferation.

One such compound is the multikinase inhibitor sorafenib. Sorafenib (Nexavar, BAY 43-9006) is a bis-aryl urea which inhibits receptor tyrosine kinases (RTKs), especially the vascular endothelial growth factor receptors (VEGFR)-1/-2/-3, the platelet-derived growth factor receptors (PDGFR)-α/-β, Flt-3 and c-KIT. Importantly, the compound was initially developed to target the MAPK pathway, and inhibits CRAF or BRAF with high affinity [10]. Furthermore, sorafenibis capable of inducing apoptosis independently of MAPK pathway inhibition by down regulation of the anti-apoptotic protein myeloid cell leukemia 1 (Mcl-1) [10]. Currently, the drug is approved for the management of metastatic renal cell carcinoma, thyroid cancer and hepatocellular carcinoma in Europe and the U.S. [11,12]. In UC, sorafenib has been tested both as a single agent and in combination with conventional cisplatin-based chemotherapy [13,14,15]. However, clinical results have been disappointing showing at best modest activity of sorafenib in treated patients.

In this context, our study aimed to explore by which mechanisms UC cells may evade the growth-inhibitory and pro-apoptotic effects of sorafenib.

## 2. Results and Discussion

### 2.1. Receptor Tyrosine Kinases Targeted by Sorafenib Are Weakly Expressed in Urothelial Cancer Cell Lines (UCCs)

We first determined the mRNA expression status of the established sorafenib targets VEGFR1, VEGFR2, PDGFR-α, PDGFR-β and cKIT. mRNA expression status of these RTKs was determined in 17 UCCs compared to eight normal uroepithelial controls (NUCs). Human umbilical vein endothelial cells (HUVEC), human fibroblasts (VHF1), and the HEK293 cell line, respectively, served as positive controls.

**Figure 1:**
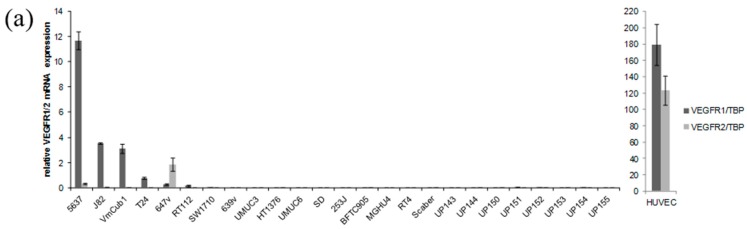

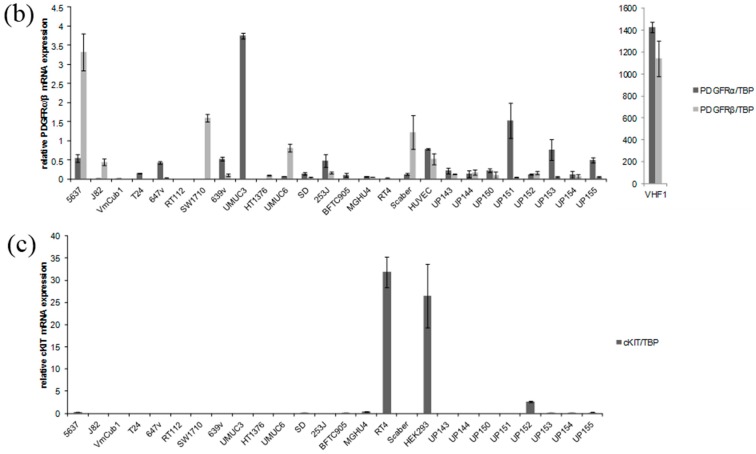
mRNA expression profiles of receptor tyrosine kinases. (**a**) Vascular endothelial growth factor receptor 1 (VEGFR1) and VEGFR2; (**b**) Platelet-derived growth factor receptorα (PDGFRα) and PDGFRβ; and (**c**) cKIT in bladder cancer cell lines, normal urothelial cells and the positive controls HUVEC (**a**), VHF1 (**b**), HEK293 (**c**). All values were measured by quantitative RT-PCR (qRT-PCR) and values were adjusted to TBP mRNA. Mean values of two independent measurements and according standard deviation are shown.

VEGFR1 was detected in 6/17 and VEGFR2 in 2/17 UCCs, but in none of the NUCs (Figure 1a). However, in all UCCs, mRNA expression of both receptors was more than a magnitude lower than in normal endothelial cells (HUVEC). PDGFRα and PDGFRβ mRNAs were both detectable in almost all UCCs as well as in NUCs (Figure 1b). However, compared to normal fibroblasts (VHF1), expression levels in UCCs and NUCs were very low. The mRNA for the stem cell factor receptor cKIT was only detectable in 1 UCC in which its expression level was comparable to the positive control HEK 293, a cell line from embryonic kidney, and in one normal urothelial cell culture (Figure 1c).

Efficacy of sorafenib treatment depends, amongst others, critically on its inhibition of MAPK signaling [16,17]. In cancer cells, this could be achieved directly by inhibition of RAF kinases in the pathway or indirectly, by inhibition of RTKs. Both effects may contribute to the efficacy of sorafenib in hepatocellular and renal cell carcinomas. In UC, various receptor tyrosine kinases are commonly overexpressed or activated by oncogenic mutations [5,6,7,8,9,18], but there is little evidence for strong expression of the RTKs targeted by sorafenib, derived mostly from smaller immunohistochemical studies. Overexpression of VEGF, a ligand of the receptor tyrosine kinases VEGFR1 and VEGFR2, has been reported in a fraction of UCs [18,19] and two papers have reported more frequent expression of VEGFR1 and VEGFR2 in UCs than in normal urothelial tissue [6,18]. Expression of c-KIT appears even less common. In a screening of various tumor entities, only 15% of UCs were c-KIT positive [20]. A statistical significant overexpression of VEGFR1 mRNA in UC compared to benign controls was detected in published microarray-data available at the Oncomine platform [18]. However, we could not confirm this finding in another large published microarray [21]. To our knowledge, expression status of PDGFRα and PDGFRβ has not yet been specifically investigated. Given the huge differences between expression levels of *VEGFR* and *PDGFR* genes in endothelial and fibroblastic cells compared to urothelial cells revealed by our qRT-PCR analysis, a major confounding factor in such studies will be the variable admixture of stromal cells to the analyzed tumor tissues.

According to publicly available expression data, BRAF and CRAF are robustly expressed in UCCs, benign and malignant urothelial tissues [21,22,23], as in almost all other epithelial tissues and cancers. In summary, therefore, our data on the UCCs and the results of other published studies suggest that the major targets of sorafenib in urothelial cancer cells should be RAF kinases.

### 2.2. Sorafenib Treatment may Result in Both Increased and Decreased Cell Viability Depending on Dosage

**Figure 2:**
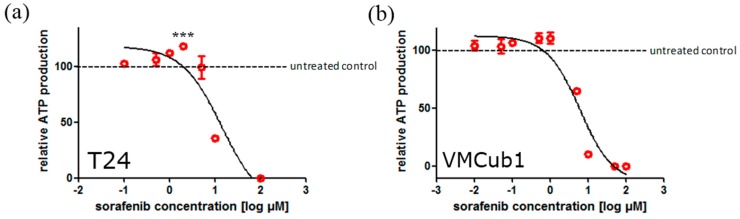
Dose-dependent effect of sorafenib on cell viability as measured by total ATP levels in (**a**) T24 and (**b**) VMCub1 cells. In T24 cells, a statistical significant increase of cell viability was observed at a concentration of 2 µM sorafenib (*** *p* < 0.05, Dunnet’s multiple comparison test).

Next, we determined the mean inhibitory concentration of sorafenib in nine UCCs (T24, 647v, SW1710, VMCub1, J82, 5637, UM-UC-3, SD, 639v) and two independent cultures of NUCs. The cell lines were chosen to represent the diverging mRNA expression patterns of potential sorafenib targets described above and to cover a large spectrum of the disease subtypes, with the exception of low grade papillary cancers. Sorafenib impaired cell proliferation [as measured by total adenosine triphosphate (ATP)] in a dose-dependent manner. Mean half maximal inhibitory concentrations (IC_50_) values after 72 h of sorafenib treatment in the UCCs ranged from 4.6 to 18 µM, whereas NUCs were much more sensitive (0.5 and 1.6 µM) (Table 1). Of note, low sorafenib doses did not result in a decrease but rather an increase of cell viability (Figure 2).

Our data on the UCCs are in accordance with a previous study on the impact of sorafenib treatment on three UCCs (T24, RT4, J82) [24]. These authors also observed an increase in cell viability along with a stimulation of cell migration and proliferation with low doses of sorafenib (<1 µM). It is possible that these effects are brought about by sorafenib promoting the dimerization of RAF proteins and thereby paradoxically activating downstream targets of RAF proteins, as described in other cell types [25]. Sorafenib effects on normal urothelial cells have not been investigated previously. Their exquisite sensitivity to sorafenib may derive from their strict dependence on growth factor signaling and MAPK activation for proliferation [26,27], which may be relaxed in UCCs with proximal defects in cell cycle regulation [3].

**Table 1:** Mean half maximal inhibitory concentrations (IC_50_) values (cell viability) of urothelial cancer cell lines (UCCs) and normal uroepithelial controls (UP199, UP202) after 72 h of sorafenib treatment as determined by total adenosine triphosphate (ATP) levels.

Cell Line	Sorafenib IC_50_ (µM)
T24	18.0
647v	16.9
SW1710	13.3
VMCub1	8.4
J82	8.1
5637	7.7
UM-UC-3	6.7
SD	5.1
639v	4.6
UP199	1.6
UP202	0.5

**Figure 3:**
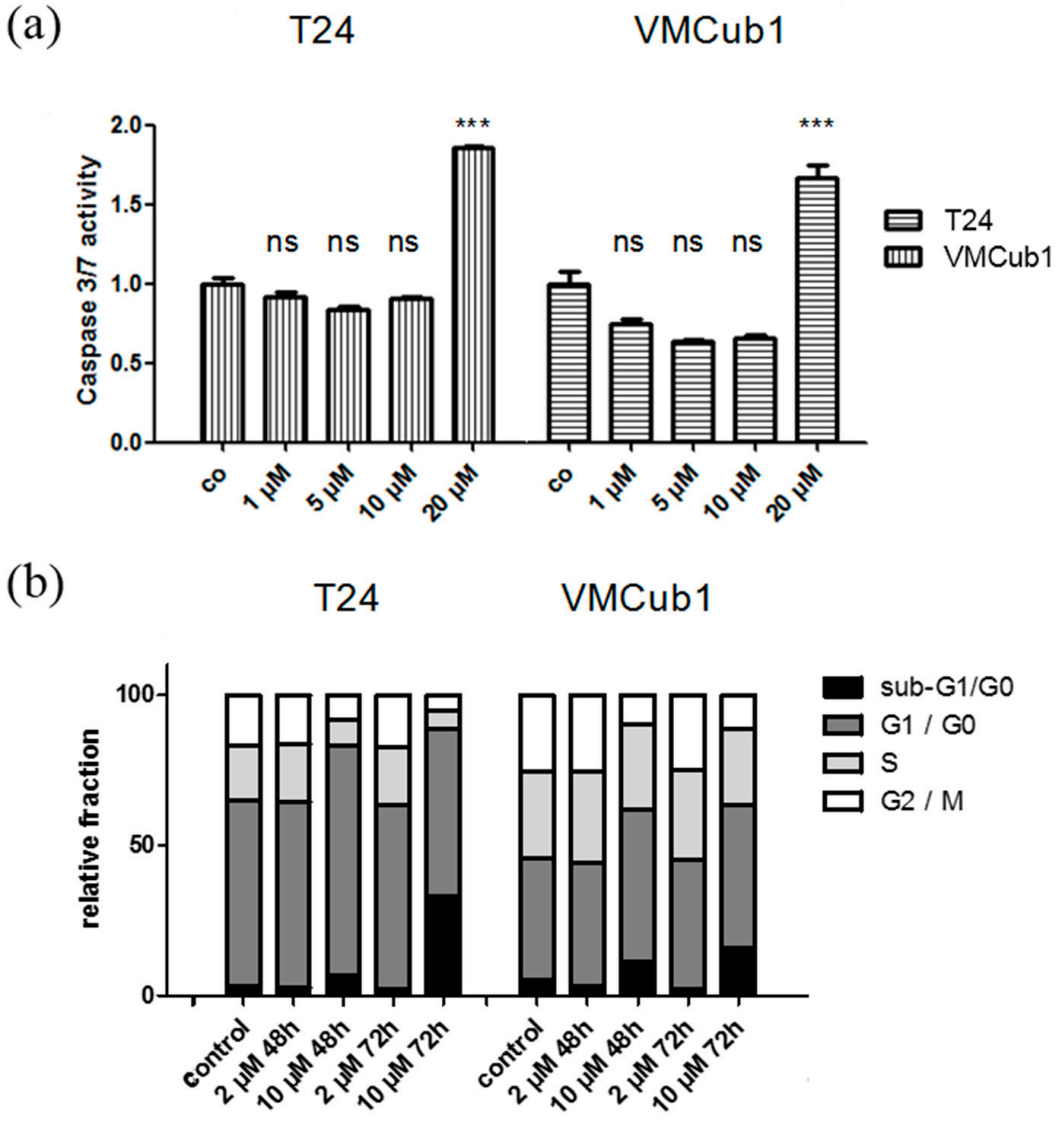
(**a**) Caspase 3/7 activity after sorafenib treatment with different concentrations for 24 h compared to untreated controls (co: control, set as 1). A significant increase in caspase 3/7 activity was noted in both T24 and VMCub1 cells at a concentration of 20 µM. (Bonferroni’s posttest, *** *p* < 0.001, ns: not significant); and (**b**) Changes in cell cycle distribution and amount of apoptotic cells (as sub-G1 fraction) after sorafenib treatment (2 and 10 µM, 48 and 72 h).

Subsequently, we investigated which mechanisms are responsible for decreased viability of UCCs after sorafenib treatment by measurement of caspase 3/7 activity, cell cycle distribution, and immunostaining of Ki-67. For these experiments, two representative cell lines, T24 with a high IC_50_ (18.0 µM) and VMCub1 with a lower IC_50_ (8.4 µM) were selected. In both cell lines, increased caspase 3/7 activity was observed only at 20 µM sorafenib (Figure 3a), even though their IC_50_ in VMCub1 was much lower. Sorafenib treatment at 2 µM did not detectably change cell cycle distribution, as measured by flow cytometry, either after 48 or 72 h of treatment. At concentrations from 10 µM, increases in the G1 fraction and the sub-G1 fraction were observed, indicative of G1-arrest and apoptosis (Figure 3b). Ki-67 staining was likewise decreased at 10 µM sorafenib (data not shown). In summary, these findings suggest that sorafenib at high concentrations induces predominantly G1-arrest and subsequently apoptosis.

### 2.3. MAPK Signaling after Treatment with Sorafenib

**Figure 4:**
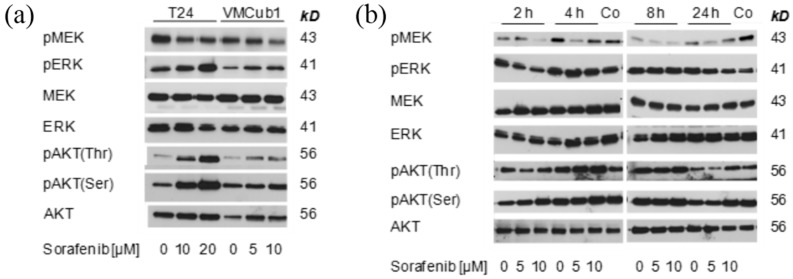
(**a**) Effect of sorafenib on phosphorylation of ERK, MEK and AKT in T24 and VMCub1 cell lines after 24 h treatment; and (**b**) Effect of sorafenib on phosphorylation of ERK, MEK and AKT in VMCub1 cells over time. The lanes marked “Co” contain extracts from T24 cells treated with 20 µM sorafenib for 24 h as positive controls. Note that the same extracts were analyzed on two different gels to avoid overlay by residual signals during the analysis of phosphoproteins.

To characterize the effect of sorafenib on MAPK signaling in UCCs, we investigated the effect of sorafenib on phosphorylation of ERK and MEK in T24 and VMCub1 cells. After 24 h of treatment, MEK phosphorylation was diminished, especially at 10 µM sorafenib in VMCub1, but ERK phosphorylation was rather increased in T24 cells (Figure 4A). As MAPK pathway inhibition may result in compensatory increases in PI3K pathway signaling, phosphorylation of AKT at Ser473 and Thr308 was investigated in the same experiment. Increased phosphorylation at both sites was observed in T24, with at most a slight increase in VMCub1 (Figure 4A).

Since a measurement at a single time point might be misleading, a time course of sorafenib effects on MEK, ERK and AKT phosphorylation was performed in VMCub1. This experiment confirmed that even in this relatively sensitive cell line, no complete suppression of ERK phosphorylation could be achieved over the course of 24 h; moreover, high concentrations of the compound in particular tended to increase AKT phosphorylation (Figure 4B).

Taken together, these results demonstrate that sorafenib inhibits MAPK signaling only to a limited degree. Most strikingly, treatment of T24 cells even with high concentrations of the compound had little effect on pathway activity and may even elicit compensatory effects through AKT phosphorylation. As T24 cells carry a known activating HRAS mutation, these resultsmay be attributed to the so called “RAF inhibitor paradox” [25,28]. Even though RAS proteins are upstream activators of RAF proteins, RAF inhibitors are poorly active and can even exert paradoxical effects in cell lines with RAS mutations [25]. Of note, in another study investigating sorafenib treatment of UCCs, efficacy of sorafenib was also lower in T24 cells compared to two other cell lines [24]. Moreover, a more detailed analysis in the most sensitive cell line, VMCub1, revealed that sorafenib does not result in a sustained repression of the pathway. These effects are likely to result from physiological feedback effects within the MAPK signaling pathway [29], which contribute to the RAF inhibitor paradox, but also occur during normal responses to growth factors. Such feedback effects might also underlie the paradoxical stimulation of cell viability by low concentrations of sorafenib seen by us and others [22]. In addition, sorafenib effects may be mitigated by crosstalk between the MAPK and the PI3K-pathway [30]. In our experiments, this crosstalk was evident by increased AKT phosphorylation particularly in T24 cells and may contribute to the low sensitivity of this cell line to RAF inhibition.

### 2.4. An Anti-Apoptotic State of UCCs Hampers Efficient Activation of Intrinsic Apoptosis

**Figure 5:**
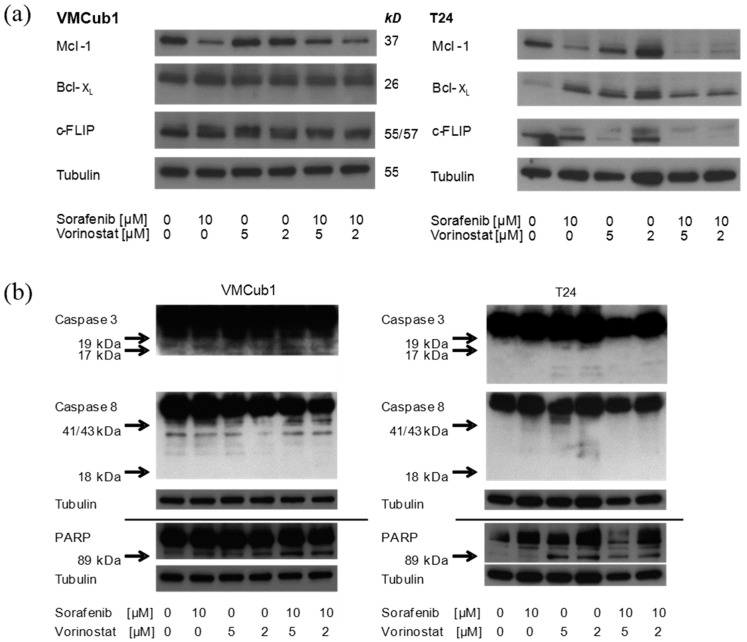

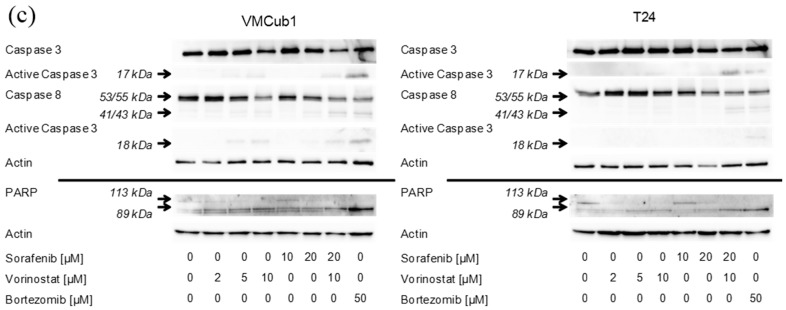
(**a**) Effects of sorafenib, vorinostat, and the combination of both compounds on anti-apoptotic factors myeloid cell leukemia 1 (Mcl-1), B-cell lymphoma-X_L_ (Bcl-X_L_), and c-FLIP. α-Tubulin was detected as a loading control. Cells were treated with sorafenib and/or vorinostat for 24 h with the indicated dosages of each compound. α-Tubulin was detected as a loading control; (**b**) Effects of sorafenib, vorinostat, and the combination of both compounds on cleavage of caspase 3, caspase 8 and poly(ADP)-ribose polymerase (PARP). Arrows indicate the expected positions of cleaved products (the blots are deliberately overexposed to visualize these). α-Tubulin was detected as a loading control. Cells were treated with sorafenib and/or vorinostat for 24 h with the indicated dosages of each compound; and (**c**) Effects of sorafenib, vorinostat, and the combination of both compounds (positive control) on cleavage of caspase 3, caspase 8 and PARP using additional anti-bodies against cleaved proteins. Bortezomib was used as positive control as described in [43]. Arrows indicate the expected positions of cleaved products. α-Tubulin was detected as a loading control. Cells were treated with bortezomib, sorafenib, and/or vorinostat for 24 h using the indicated concentrations of each compound.

Induction of apoptosis is a mainstay of sorafenib action in a number of cancers, e.g., hepatocellular carcinoma, endometrial cancer, and myeloma [16,31,32]. Therefore, we investigated potential mechanisms impairing the efficacy of sorafenib on activation of apoptosis in UCCs. In particular, the pro-apoptotic effect of sorafenib has been attributed to the down regulation of the anti-apoptotic protein Mcl-1, a non-canonical target of MAPK signaling [33]. In UCCs as well, sorafenib treatment (10 µM, 24 h) resulted in a decrease of Mcl-1 protein expression in all investigated UCCs as well as in NUCs (data not shown). In contrast, two further important anti-apoptotic factors, B-cell lymphoma-X_L_ (Bcl-X_L_), a member of the B-cell lymphoma 2 (Bcl-2) protein family acting in a similar fashion as Mcl1 [34,35,36] and c-FLIP, an anti-apoptotic protein opposing death receptor-driven apoptosis [37,38] were not consistently affected (Figure 5a). In order to concomitantly down-regulate Bcl-X_L_ and c-FLIP, VMCub1 and T24 cells were treated additionally with the histone deacetylase inhibitor SAHA (vorinostat), which has been described to synergize with sorafenib in inducing apoptosis by targeting both c-FLIP and Bcl-X_L_ [39]. Interestingly, Bcl-X_L_ was not diminished following treatment with SAHA, which down-regulates the protein in other cancers [40,41,42]. The combination of vorinostat and sorafenib likewise only diminished Mcl-1, but not Bcl-X_L_ (Figure 5a). Effects of vorinostat on c-FLIP were observed only in T24 cells, whereas the protein remained strongly expressed in VMCub1 cells. In line with these observations, following treatment with sorafenib (10 µM) alone, vorinostat alone (2 or 5 µM) or their combination, very limited cleavage of caspase 3, caspase 8 or poly(ADP)-ribose polymerase (PARP) was observed (Figure 5b,c). Accordingly, dose-response evaluation of combined sorafenib and vorinostat treatments of UCCs yielded, at most, additive effects (data not shown).

Considered together with the results from the cell cycle analysis and caspase activity assays, these data indicate that sorafenib induces only a limited degree of apoptosis in UCCs, at comparatively high concentrations and following cell cycle arrest. We have made analogous observations in our previous analysis of vorinostat action in UC, where apoptosis was induced, but only at relatively high concentrations and in a delayed manner [39]. The combination of both compounds has been reported to act synergistically on various cancer cell lines, e.g., hepatocellular, renal, sarcoma and pancreatic cancer, albeit by often complex mechanisms [39,44,45,46,47]. Importantly, in these cell lines, the compounds induce and/or activate the death receptor CD95 (Fas) with subsequent caspase 8 cleavage and down-regulate the anti-apoptotic proteins Mcl-1 and c-FLIP as well as other proteins of the Bcl-2 family. Of these effects, only Mcl-1 down regulation by sorafenib was well detectable in the UC cell lines. Of note, inactivation of CD95 signaling is well established in UCC [48,49]. Likewise, the level of c-FLIP present in UCC is limiting for apoptosis [50].

**Figure 6:**
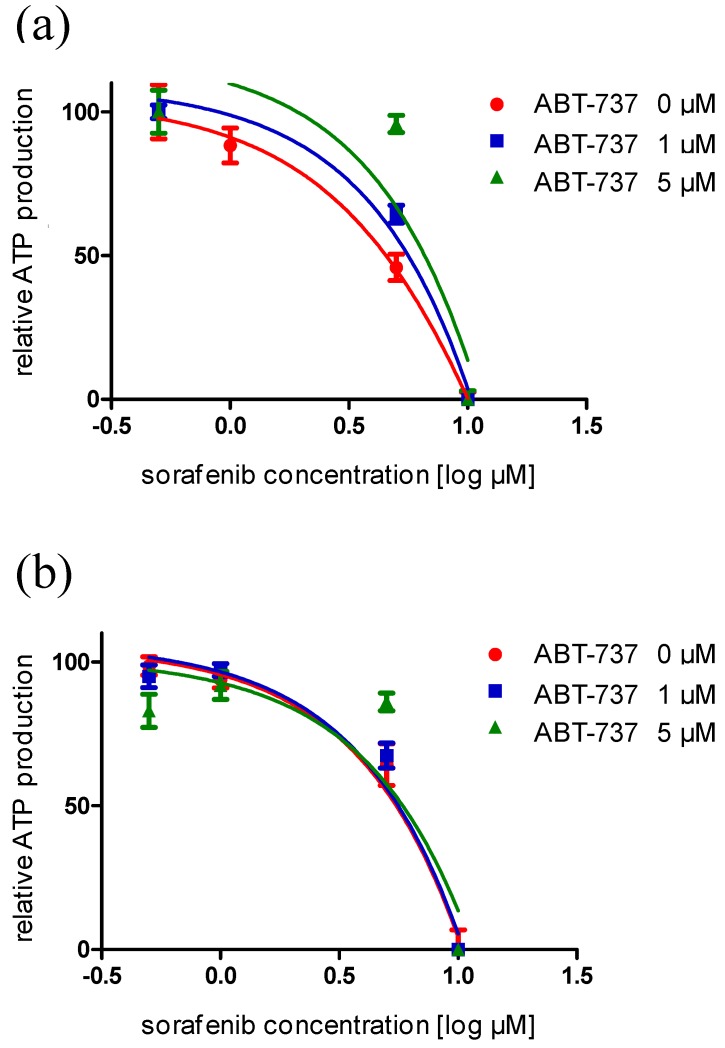
Dose-dependency of sorafenib effects on cell viability in combination with the Bcl-2 inhibitor ABT-737 as measured by total ATP levels, shown exemplarily in T24 cells. Cells were treated with both compounds over (**a**) 24 and (**b**) 72 h.

Specific inhibition of Bcl-2 family proteins often potentiates the apoptotic effects of sorafenib treatment [51,52]. In particular, treatment with ABT-737, an inhibitor of many Bcl-2 family proteins apart from Mcl1, including Bcl-X_L_, synergizes with sorafenib to enhance apoptosis in hepatoma cells [51,53,54]. However, in dose-response evaluation, no evidence of synergy between ABT-737 and sorafenib was obtained with UCC cell lines (Figure 6).

Cumulatively, these findings point towards an anti-apoptotic state of UCCs that may be caused by several mechanisms, in addition to expression of anti-apoptotic proteins like Mcl-1, Bcl-X_L_ and c-FLIP [50]. Evidently, these mechanisms need to be investigated more deeply, as they should be relevant not only in the response to sorafenib (and vorinostat), but also to other targeted therapies and perhaps even cytotoxic chemotherapy.

## 3. Experimental Section

### 3.1. Cell Lines and Cell Cultivation Procedures

All bladder cancer cell lines were cultured in DMEM GlutaMAX-I (Gibco Life Technologies, Karlsruhe, Germany), supplemented with 10% fetal calf serum, 100 U/mL penicillin and 100 μg/mL streptomycin. Primary urothelial cells were prepared from ureters after nephrectomy and were routinely maintained in keratinocyte serum-free medium (KSFM, Gibco Life Technologies, Karlsruhe, Germany) supplemented with 50 mg/mL bovine pituitary extract and 5 ng/mL epidermal growth factor as described. Human umbilical vein endothelial cells (HUVEC) and HEK293 cells, respectively, were kindly provided by Dr. G. Kögler (Institute for Transplantation Diagnostics and Cell Therapeutics, Heinrich Heine University Medical Center, Duesseldorf, Germany) and Dr. V. Kolb-Bachofen (Institute of Molecular Medicine, Research Group Immunobiology, Heinrich-Heine-University, Duesseldorf, Germany). Normal human foreskin fibroblasts (VHF) were cultured as described [55].

### 3.2. Determination of Cell Viability, Mean IC_50_ and Caspase 3/7 Activity

Cell viability and apoptotic activity was determined via total ATP by the Cell Titer-Glo Luminescent Cell Viability Assay (Promega, Mannheim, Germany) and Caspase-Glo 3/7 Assay (Promega, Mannheim, Germany), respectively, according to the manufacturer’s protocol in a plate reader luminometer (Wallac Victor 2; Perkin-ElmerLife Sciences, Boston, MA, USA). For determination of IC_50_ by this method cells were seeded in 96-well plates at 1.5 × 10^4^ cells per well. The next day, cells at 30%–40% confluency were incubated with various concentrations of sorafenib (0.01–100 µM) in quadruplicates for 72 h with daily medium changes. Dose-response curves and IC_50_ values were approximated by non-linear regression analysis, differences in caspase 3/7 activity at different sorafenib concentrations were compared to untreated controls by Bonferroni’s posttest (GraphPad Prism v5.01; GraphPad Software, La Jolla, CA, USA).

### 3.3. Cell Cycle Analysis by Flow Cytometry

For fluorescence-activated cell sorting (FACS) analysis, cells were harvested by trypsinization, washed with PBS, then stained with 50 μg/mL propidium iodide solution containing 0.1% triton X-100 and sodium citrate as described [56] and were analyzed for cell cycle distributions using a FACS Calibur instrument (Becton Dickinson, Heidelberg, Germany). Cell cycle profiles were analyzed using CellQuest Software (Becton Dickinson, Heidelberg, Germany).

### 3.4. RNA Extraction and Analysis

Total RNA was isolated using the Qiagen RNeasy MiniKit (Qiagen, Hilden, Germany) according to the manufacturer’s protocol. Synthesis of cDNA was performed using SuperScriptII reverse transcriptase (Invitrogen, Darmstadt, Germany) with mixed oligo-dT and random primers, according to the manufacturer’s protocol.

Real-time RT-PCR assays were performed using a LightCycler II (Roche, Mannheim, Germany). For VEGFR1, VEGFR2, PDGFRα, PDGFRβ, cKIT mRNA as well as the reference gene TBP specific a LightCycler II (Roche, Mannheim, Germany). QuantiTect primer assays (Qiagen, Hilden, Germany) with the QuantiTect SYBR Green PCR Kit (Qiagen, Hilden, Germany) were applied. All measurements were performed in at least duplicates; variance was less than 10%.

### 3.5. Western Blot Analysis

Whole cell lysates were prepared and quantified as previously described [26]. Equal amounts (10–15 µg) of protein were separated by SDS-polyacrylamide gel electrophoresis (Precise Protein Gel; Thermo Scientific, Rockford, IL, USA) and transferred to an Immobilon-P membrane (Millipore Corp., Bedford, MA, USA). The membranes were then blocked with 5% nonfat milk powder or BSA and 0.1% Tween 20 in Tris-buffered saline. Samples were successively probed with primary antibodies against pERK (Thr202/Tyr204, #4376), pMEK (Ser217/221, #9121), pAkt (Ser473, #4058), pAkt (Thr308, #4056), MEK (#9122), AKT (#9272), Mcl-1 (#4572), caspase-3 (#9665), active caspase-3 (#9661), active caspase-8 (#9496), PARP (#9532) (Cell Signaling Technology, Danvers, MA, USA), ERK (#06-182; Upstate via Merck, Darmstadt, Germany), Bcl-X_L_ (#556361; BD Pharmingen, Heidelberg, Germany), caspase 8 (#ALX-804-242), c-FLIP (#ALX-804-428) (Alexis/Enzo Life Science, Lörrach, Germany), caspase-3 (AF-605-NA; R&D Systems, Minneapolis, MN, USA), Actin (A2228; Sigma–Aldrich, St. Louis, MO, USA) and caspase-8 (12F5; kindly provided by Klaus Schulze-Osthoff, University of Tübingen). Protocols on the individual antibody dilution and the individual method of antibody blocking are available upon request. In general, incubation was performed overnight at 4 °C in 1% nonfat milk powder or 5% BSA and 0.1% Tween 20 in Tris-buffered saline. As a loading control, anti-α-tubulin B-5-1-2 (Sigma, St. Louis, MO, USA) was used at a 1:50,000 dilution for 60 min at room temperature. HRP-conjugated rabbit-anti-mouse antibody (1:200,000) or goat-anti-rabbit antibody (1:200,000) (Santa Cruz Biotechnology, Heidelberg, Germany) were used as secondary antibodies. Bands were visualized using the ECL advanced chemoluminescence kit (GE Healthcare, Piscataway, NJ, USA). More concentrated secondary antibodies (1:5000 or 1:20,000, respectively) were required with the Quantum Western Bright chemoluminescence kit (Biozym, Hessisch-Oldendorf, Germany) used in some experiments.

### 3.6. Compounds

Sorafenib (Nexavar; Bayer, Leverkusen, Germany), ABT-737 (Selleckchem, Munich, Germany) and suberoylanilidehydroxamide acid (SAHA; Cayman, Ann Arbor, MI, USA) were dissolved in dimethyl sulphoxide (DMSO) as 10 mM stock solutions and stored at −20 °C until use. In all cell culture experiments, DMSO concentrations up to 0.2% were applied since UC cell viability becomes impaired only by DMSO concentrations ≥0.5% (data not shown).

## 4. Conclusions

In summary, the efficacy of sorafenib in urothelial cancer cells appeared to be impeded by several factors, including lack of targets, inherent or oncogene-enhanced robustness of MAPK signaling, likely crosstalk with other pathways such as PI3K/AKT signaling, and an anti-apoptotic state. From our observations and those of others [22] in cell lines, sorafenib action in UC tissues would have to rely solely on its anti-angiogenic effects and exert few or even counterproductive effects on cancer cells themselves, other than in hepatocellular carcinoma and renal cell carcinoma, where the drug is efficacious. Our results therefore may help to explain the failure of clinical trials with this compound in UC. Moreover, our results may be of interest beyond sorafenib in particular in that the mechanisms limiting its action as a RAF inhibitor on UC cells may also be relevant for other drugs targeting signal transduction pathways in UC. As recent genomic studies [57,58] have deemed mutations in many UC “actionable”, these mechanisms require further detailed analysis. In particular, the mechanisms limiting apoptosis in urothelial carcinoma cells may need comprehensive characterization.
